# Embedding Active Pedagogies within Pre-Service Teacher Education: Implementation Considerations and Recommendations

**DOI:** 10.3390/children7110207

**Published:** 2020-11-02

**Authors:** Natalie Lander, Emiliano Mazzoli, Samuel Cassar, Naomi Symington, Jo Salmon

**Affiliations:** 1 Faculty of Arts and Education, Deakin University, Geelong, VIC 3220, Australia; n.symington@deakin.edu.au; 2 Institute of Physical Activity and Nutrition (IPAN), Faculty of Health, Deakin University, Geelong, VIC 3220, Australia; e.mazzoli@deakin.edu.au (E.M.); s.cassar@deakin.edu.au (S.C.); jo.salmon@deakin.edu.au (J.S.)

**Keywords:** physical activity, mixed methods, RE-AIM, education, student, implementation, scalability, evidence-based practice

## Abstract

The physical activity levels of children in Australia are critically low and correlate with reduced academic achievement and poor health outcomes. Schools provide an ideal setting for physical activity interventions to help children move more. Instead of targeting in-service teachers, this study embedded an evidence-based active pedagogy program called Transform-Ed! into pre-service teacher education. Pre/post surveys and post-program interviews and focus group discussions were conducted with key stakeholders (*n* = 5), lecturers (*n* = 6), and pre-service teachers (*n* = 274) involved with the 12-week program. The design, implementation, and evaluation of the study were systematically guided by all five dimensions of Glasgow and colleagues’ RE-AIM (reach, effectiveness, adoption, implementation, and maintenance) framework. Linear mixed models, descriptive analysis and a framework approach were used to analyse the data. Significant improvements were observed in pre-service teachers’ willingness, confidence, and competence to implement physically active pedagogic strategies following the intervention. Pre-service teacher perceived effectiveness of such strategies on student outcomes also significantly increased and perceived barriers decreased. High adherence was consistently reported and the program was maintained after completion of the implementation trial by all lecturers. Four key themes spanning multiple dimensions and participant levels informed recommendations for program scalability: an “inter-systemic approach”, a “co-design” approach, “embedded in professional practice”, and “evidence of impact” on teacher practice. Anchored in real-world settings and tethered by implementation science, Transform-Ed! could have the potential to advance the teaching capability of teachers, and transform the learning experience and physical and academic outcomes of primary school students.

## 1. Introduction

It is well known that physical activity is vital for the health and development of children [1], while high levels of sedentary time is linked to negative psychosocial and physical health outcomes [2,3]. There is growing evidence that physical activity improves classroom concentration, behaviour, cognitive function and academic achievement [4,5,6]. However, students spend 70% of the school day sitting [7] and globally, over 60% of children fail to meet international guidelines of an hour of moderate-to-vigorous physical activity a day [8]. In Australia, less than two in ten children meet physical activity guidelines [9]. As children spend a large proportion of their week at school, it is an ideal setting for interventions that increase activity levels [10], such as via active lessons, active breaks, and active environments [11].

A recent systematic review of classroom-based physical activity interventions highlighted how they can be a low-cost, practical and time-sensitive way to increase physical activity throughout the school day [12]. However, schools still face major challenges in adopting and implementing change [13]. For interventions to affect children at a population level, they need to be executed in real-world settings, rather than under controlled research conditions, and at scale [14].

Transform-Us! is one example of a successful school-based physical activity intervention. Based on social cognitive theory [15], behavioural choice theory [16], and ecological systems theory [17], the program aims to break up prolonged sitting and promote movement using innovative behavioural and pedagogical strategies across the school day [18]. The program was proven to be effective in a cluster randomised controlled trial of seven- to nine-year-old children in 20 Victorian primary schools [19,20]. It has since been adapted for real-world implementation at scale and is currently available to all primary schools in Victoria (https://transformus.com.au/).

Implementation science examines how evidence-based interventions can be applied in routine practice [21]. The RE-AIM (reach, effectiveness, adoption, implementation, and maintenance) framework was developed by Glasgow and colleagues [22] to aid reporting of health interventions and address the slow translation of scientific advances into public health impact [23]. Although currently limited, the application of implementation science in school-based physical activity interventions has led to a better understanding of the factors influencing effective uptake [24,25]. For example, a lack of time and teacher overload are consistently mentioned as barriers to the implementation of programs. Further, the quality of training and teacher efficacy often correlates with implementation success [25,26,27]. We know the quality of pre-service training has a significant impact on learning and teaching outcomes more broadly, but little is known about the effectiveness of embedding physical activity interventions in pre-service teacher education [28,29]. Focusing interventions on pre-service teacher training is a potentially important and understudied avenue for intervention success and ‘scalability’ [30].

Pre-service or initial teacher education should prepare graduates for classroom teaching with evidence-based content knowledge and a solid understanding of teaching practices proven to optimise student learning [31]. Indeed, pre-service teacher education is pivotal in developing effective teaching approaches and positive student outcomes [32]. Despite its importance, there has been some critique that current initial teacher education programs are disconnected from practice and are not informed by evidence, subsequently they may inadequately prepare new graduates for teaching [29,31]. Despite these criticisms, consensus exists on some variables beneficial for practicing teachers, such as, perhaps most importantly, teachers who have more self-efficacy and competence tend to be more effective [33,34,35]. Thus, integrating evidence-based physical activity programs early in pre-service teacher education may prove to be not only an effective way to upskill future generations of teachers but also a powerful pathway for implementing school-based physical activity interventions.

In 2018, a pilot study demonstrated the feasibility of Transform-Ed!, an evidence-based active pedagogy program, that was embedded into a core first-year unit of the Bachelor of Education (Primary) degree at Deakin University [36]. The next step for Transform-Ed! is to evaluate the effectiveness of implementation [37]. The application of implementation models and theories may be one way to address the research-practice gap and better understand challenges faced by organisations [38,39]. The present study may be the first to use implementation theory to understand the potential of pre-service teacher training to support interventions in real-world school systems. The primary purpose of this research was to investigate the reach, effectiveness, adoption, adaption, implementation and maintenance of Transform-Ed! across the first year of an undergraduate teacher education unit using the RE-AIM framework [22]. A further aim was to provide guidance for the potential scale-up of the program.

## 2. Materials and Methods

Informed by the successful Transform-Us! program, Transform-Ed! aimed to educate and upskill pre-service teachers in (i) planning and delivering active teaching, (ii) creating active environments, and (iii) encouraging active families. Examples of active pedagogic strategies include physically active academic lessons, physically active breaks from prolonged sitting, health-based curriculum content, alterations to the classroom to enable activity, encouraging activity at recess and lunchtime, and physically active homework. The research team and key stakeholders at the university (i.e., head of school, course directors, unit chairs, and lecturers) co-created the Transform-Ed! curriculum to align it with the priorities, structure, objectives and desired outcomes of pre-service teacher education [36].

### 2.1. Conceptual and Theoretical Frameworks

**Figure 1:**
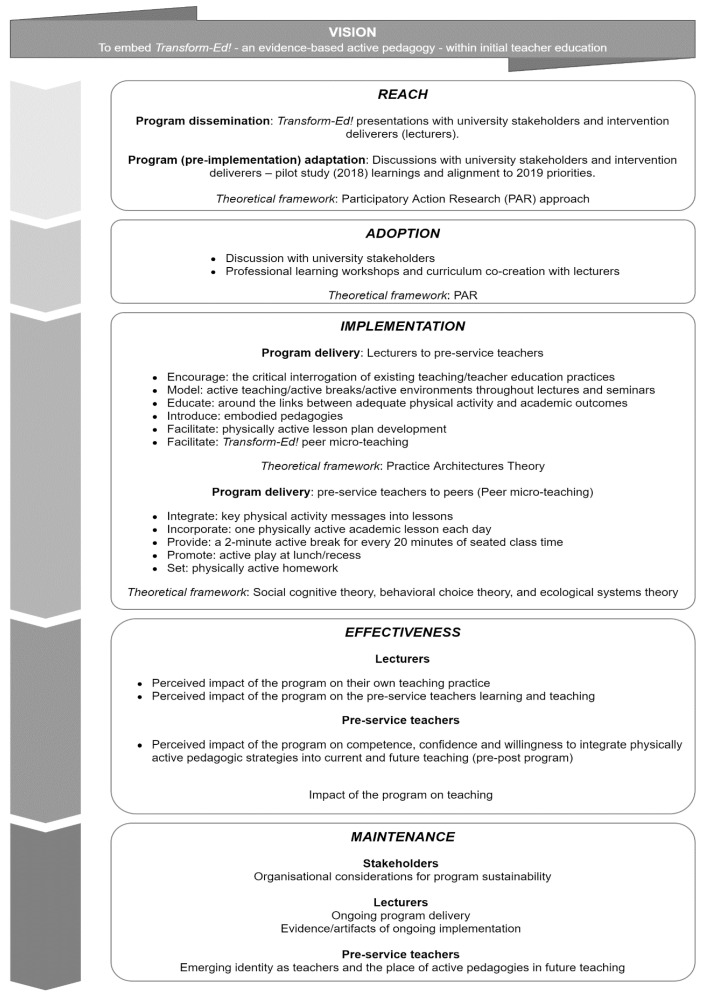
Conceptual overview of the trial implementation.

The present implementation trial draws from multiple evidence-based theories and frameworks, as shown in Figure 1 and discussed below.

#### 2.1.1. RE-AIM

First and foremost RE-AIM [22] was the overarching framework comprehensively used to design, implement and evaluate Transform-Ed!. The RE-AIM framework is designed to enhance the quality, speed, and public health impact of efforts to translate research into practice [22].

#### 2.1.2. Participatory Action Research

Participatory action research [40,41] was also heavily referenced in Transform-Ed!. Participatory action research is an approach to research that emphasizes participation and action and has played a pivotal role in educational change [40,42]. In the current research, the intention was that key stakeholders (i.e., senior academics) were active contributors to the research. Senior academics are the organisational decision-makers; therefore, their participation from the inception of the program was strategically important in relation to the inclusion of Transform-Ed! in the Bachelor of Education (primary) course. Senior academics were involved in the formative stages of program development (i.e., the feasibility study) and in the program adaptation and refinement for the implementation trial. This included two 1-h workshops, focusing on potential barriers and facilitators to program dissemination, adaptation and adoption (see Figure 1), as well as post-program reflections. Due to the pivotal role participatory action research (PAR) has played in the development of teachers and teaching, curriculum development and evaluation, PAR also heavily framed lecturer participation. Lecturers were invited to participate in pre-program dissemination and professional learning, and were active contributors in program adaptation and re-development, curriculum and content design and program delivery (see Figure 1). This included three 2-h co-design workshops prior to the commencement of program implementation, and ongoing involvement, contribution, iteration, and refinement to program content and delivery across the 12 weeks, as well as post-program reflections.

#### 2.1.3. Practice Architecture Theory

Practice architecture theory [42] informed delivery of the intervention from lecturer to pre-service teacher (see Table 1). Specifically, lecturers used practice architecture theory to enable pre-service teachers to critically interrogate existing teaching and pre-service teacher education practices, and to create new possibilities for teaching, particularly around active pedagogies. The purpose was to facilitate the shift from student (i.e., ‘learner’) to practitioner (i.e., ‘teacher’), particularly regarding confidence and competence in delivering active pedagogies. In the present study, pre-service teachers were involved as participants (learner), receiving Transform-Ed! from the lecturer, and as change agents (teacher) delivering Transform-Ed! in peer micro-teaching.

In addition to using practice architecture theory, lecturers used the fundamental elements of embodied pedagogy and transformative education. Embodied pedagogy joins the body and mind in a physical and mental act of knowledge construction [43], which is particularly relevant to active academic lessons (see Table 1). Transformative education suggests that learning is the process of using a prior interpretation to create a revised understanding [44]. This approach is known to be effective in pre-service teacher education regarding physically active pedagogies [36]; it was used in the current study to affect change in perspective for pre-service teachers.

#### 2.1.4. Sociocultural Theory (Peer Micro-Teaching)

Peer support in learning is underpinned by the sociocultural theory of learning [45], and in this study it guided the delivery of the program from pre-service teacher to pre-service teacher (peer micro-teaching). The theory holds that learning and development can be progressed faster through social interactions [45]. In peer micro-teaching, key Transform-Ed! concepts are delivered by peers and they request constructive feedback from peers about potential improvements to their teaching technique. In the present study, the peer micro-teaching experience facilitated understanding of teaching practices that have been found to make a difference to primary school student physical activity [19,46].

### 2.2. Recruitment and Consent

**Figure 2:**
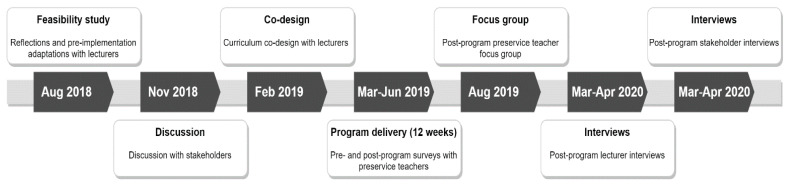
Project timeline.

Three levels of participants were recruited to the study. *Senior academics* (e.g., Head of School, Associate Head of School, Head of Teaching and Learning, Head of Research, Associate Head of Research, Course Directors, Unit Chairs, Professional Placement Coordinator) were invited to attend the pre-program presentations, re-development discussions and post-program interviews. All *lecturers* and sessional staff likely to be responsible for the delivery of the core unit in which Transform-Ed! was embedded were invited to participate in initial program dissemination (presentations) and pre-implementation adaptation workshops, curriculum co-creation sessions, Transform-Ed! delivery, a self-report adherence checklist and post-program interviews (Figure 2).

All *undergraduates* (i.e., pre-service teachers) enrolled in the Transform-Ed! unit were invited to participate in the study. Further advertisement of the study included word of mouth, direct emails, flyers, the School of Education newsletter, and the unit online platform. A plain language statement was provided to all participants via email, and signed consent to participate was required. The study was approved by University Faculty of Arts and Education–Human Ethics Advisory Group (HAE-17–207).

### 2.3. Study Design

The 12-week Transform-Ed! program was run in Trimester 1, 2019, embedded in a core curriculum unit of the undergraduate Bachelor of Education (Primary) degree. The unit also serves as an elective available to undergraduates outside of the Bachelor of Education (Primary) degree, for example Bachelor of Arts. The 12-week duration was selected to align with the length and structure of the Trimester and unit. A mixed method approach was used to evaluate the Transform-Ed! implementation trial (Table 2). The use of qualitative methods (i.e., interviews and focus group discussion) at three participant levels enhanced the usefulness of RE-AIM [23,47] by providing richness and adding depth and meaning to facilitate understanding [47]. The project timeline is described in Figure 2.

The RE-AIM Model Dimension Items Checklist (http://www.re-aim.org/wp-content/uploads/2016/09/checklistdimensions.pdf), which indicates ‘exemplar use for each RE-AIM dimension’, and the RE-AIM Criteria and Scoring Instrument (https://rtips.cancer.gov/rtips/reAimCriteria.do) were used to guide the quantitative aspects of the study (see Supplementary File S1). Each RE-AIM dimension was scrutinised separately across each participant level; however, pre-service teachers are not included in every RE-AIM dimension as some are focused on the setting or organisational aspects rather than the individual level.

As summarised in Table 2, Reach was operationalised as the count, proportion and representativeness of individuals willing to participate in the program. Effectiveness was viewed as the impact of the intervention on important individual outcomes. At the key stakeholder level, this was assessed qualitatively via examining whether the effectiveness outcomes were meaningful, provided information that helps decision-making, and were meaningful enough to make the intervention worthwhile. Perceived barriers and facilitators of program effectiveness were investigated with senior academics and lecturers. At the level of the pre-service teacher, implementation effectiveness was measured by changes in perceived competence, confidence, and willingness to deliver active pedagogies in future teaching practice, barriers and facilitators, as well as the perceived impact of the program on student outcomes. Adoption was viewed as the number, proportion and representativeness of settings and intervention deliverers willing to deliver the program, and included information on deliverers’ motivation to adopt the program. Adoption also related to understanding how the intervention could vary between settings (i.e., campuses, deliverers). At the organisation level, implementation was referred to as fidelity or adherence to the intervention including consistency of delivery. It also included adaptations to intervention content and implementation strategies. Program implementation included information on delivery by lecturers and peer micro-teaching (see Figure 1, Table 1, and Supplementary File S2). Maintenance was operationalised at the organisation level and was defined as the extent to which the program became part of routine practices and policies. It also reflected the long-term impact of the program, or sustainability.

### 2.4. Data Collection

Semi-structured individual in-depth interviews were conducted post-program with five stakeholders (senior academics) in the School of Education, through Zoom video calls (https://zoom.us/). Stakeholder interviews explored organisational or system level barriers and facilitators to program reach, adoption, implementation, effectiveness, and maintenance (see Supplementary File S3: Stakeholder interview guide). Stakeholder interviews ranged from 35 to 75 min (mean duration 49 min). Individual in-depth online interviews were also conducted with six lecturers who delivered Transform-Ed!, post program. The interviews aimed to investigate their perceptions and insights regarding challenges and enablers of all five RE-AIM domains (see Supplementary File S4: Lecturer interview guide). Lecturer interviews ranged in duration from 22 to 45 min (mean duration 34 min).

Interviews were guided by the ‘Key Considerations for Qualitative Data with RE-AIM’ (http://www.re-aim.org/qualitative-guidance-overview/#Template) and the ‘RE-AIM Elements and Qualitative Data Questions and Examples’ [47], and aimed to facilitate in-depth discussion around each of the RE-AIM dimensions. ‘Member checking’ was performed during the interviews by summarising and relaying participant information to establish accuracy [48,49]. Lecturer adherence checklists were also used as a measure of implementation (see Supplementary File S2).

To evaluate program effectiveness at the individual level, baseline and follow-up self-report surveys were completed by 274 pre-service teachers who undertook Transform-Ed!. The survey has demonstrated high levels of reliability [36]. Survey responses were based on a 5-point Likert scale (1 = strongly disagree to 5 = strongly agree). In addition, five semi-structured focus group discussions with a subsample (*n* = 30) of pre-service teachers were conducted to (i) build on survey responses, (ii) share reflections of program adoption, effectiveness, and implementation while peer micro-teaching, and (iii) discuss the perceived impact of the program on their emerging identity as teachers. Focus groups were conducted face-to-face in a meeting room at the university campuses, with six pre-service teachers per focus group. Member checking was performed during the focus groups to enhance trustworthiness [48,49]. Pre-service focus group discussions ranged in duration from 15 to 20 min (mean duration 18 min).

### 2.5. Data Analysis

Descriptive statistics were used to report on the counts and proportions related to program *reach* and *adoption*. Descriptive statistics were calculated to indicate lecturers’ self-reported adherence to the program, which was summarised by key Transform-Ed! domains (e.g., active academic lessons, active breaks, engagement with families). For each domain, a new variable indicating the average responses to the related sub-questions was generated.

All interviews and focus groups were transcribed verbatim and saved using a digital text editor (i.e., Microsoft Word 2018, Version 1806, Microsoft, Redmond, WA, USA). Participants were emailed a copy of the transcript to review interpretive accuracy. The transcripts were read, manually coded and collated into categories relevant to each domain of the RE-AIM framework. Coding reliability was conducted by two authors (N.L. and N.S.), who also completed reliability testing of two focus groups (i.e., 33% of the data). Coding decisions were compared and divergent choices were discussed until agreement was found. The remaining data analysis was conducted by N.L., and the accuracy of the coding process was verified by N.S.

**Figure 3:**
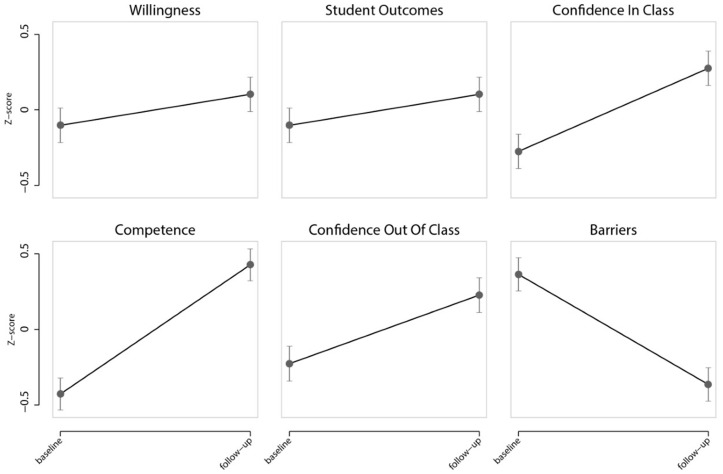
Changes in pre-service teachers’ perceptions on using physically active pedagogic strategies following Transform-Ed!.

Baseline to follow-up changes in self-reported willingness, confidence, competence, effectiveness, and barriers related to active pedagogic strategies analysed with linear mixed models clustered at the individual level and adjusted for pre-service teachers’ gender, age, university course and year level. To calculate standardised coefficients, z-scores of outcome variables and predictors were calculated prior to conducting the analyses with linear mixed models, in STATA. Obtained standardised coefficients were used to depict the intervention effects as presented in Figure 3. Standardised coefficients allow results to be compared from domains that use different scales. Considering that different domains in the survey we used had different score ranges, standardised coefficients were considered appropriate for a graphical representation. For completeness, unstandardised coefficients were also reported and full results from mixed models (including fixed effects and random effects) are available in Supplementary File S5. The sample demographic information was also calculated. These analyses were conducted using STATA^®^ 16.0 (Stata Statistical Software, Release 16, StataCorp LLC, College Station, TX, USA). To provide a measure of effect size, Cohen’s *f* ^2^ was calculated using the standardised coefficients obtained from the mixed models and following this equation:

$$f^{2}=\;\frac{\beta^{2}}{1-\beta^{2}}$$

Effects sizes were identified as small for *f*^2^ ≥ 0.02, moderate for *f*^2^ ≥ 0.15 and large for *f*^2^ ≥ 0.35.

## 3. Results

### 3.1. Participants

#### 3.1.1. Stakeholders

Eleven senior academics participated in presentations, and five participated in all aspects of data collection (i.e., the Head of School, Head of Teaching and Learning, Head of Research, Course Director and the Unit Chair). Two declined the post-program interviews due to lack of time and four did not respond to the interview invitation.

#### 3.1.2. Lecturers

Six lecturers were involved in curriculum re-development discussions and co-design (moving from feasibility to implementation). Five of these lecturers delivered Transform-Ed! to pre-service teachers.

#### 3.1.3. Undergraduates (Pre-Service Teachers)

The intervention was delivered across three Victorian campuses (one metropolitan and two rural), to 274 undergraduate pre-service teachers (i.e., 18 at Warrnambool campus, 77 at Waurn Ponds campus, 179 at Burwood campus). Of these, 258 students completed the survey [36] before and after the intervention. The majority were aged between 17 and 21 years (71%), female (76%), and were enrolled in the Bachelor of Education (Primary) (78%) in their first year (63%). Thirty pre-service teachers participated in the focus group discussions.

### 3.2. Quantitative Data

High lecturer adherence was consistently reported for transmission of content related to active lessons, active breaks and health-related curriculum (Table 3). Four of the lecturers also reported high adherence in relation to embedding content related to active environments and engaging families in their teaching material.

The results from linear mixed models showed significant improvements in pre-service teachers’ perceptions regarding their own willingness (B = 0.54, 95% CI (0.22, 0.86), *p* = 0.001), confidence (B_in class_ = 1.76, 95% CI (1.31, 2.21), *p* < 0.001; B_out of class_ = 1.72, 95% CI (1.15, 2.29), *p* < 0.001) and competence (B = 2.84, 95% CI (2.38, 3.30), *p* < 0.001) in implementing physically active pedagogic strategies, following the intervention. Moreover, the perceived *effectiveness* of such strategies on student outcomes increased (B = 1.75, 95% CI (1.32, 2.18), *p* < 0.001) and perceived barriers decreased (B = –8.25, 95% CI (–9.73, –6.77), *p* < 0.001). Baseline and follow-up standardised predicted margins of each outcome of interest are presented in Figure 3. The effect sizes of the intervention were small for pre-service teachers’ willingness (*f*^2^ = 0.04), moderate for perceived effectiveness on student outcomes (*f*^2^ = 0.27) and confidence out of class (*f*^2^= 0.26), and large in relation to confidence in class (*f*^2^ = 0.43), perceived barriers (*f*^2^ = 1.13) and competence (*f*^2^ = 2.71). Complete results of effects on pre-service teachers’ perceptions adjusted for gender, age, course, and year level are available in Supplementary File S5.

### 3.3. Qualitative Data

**Figure 4:**
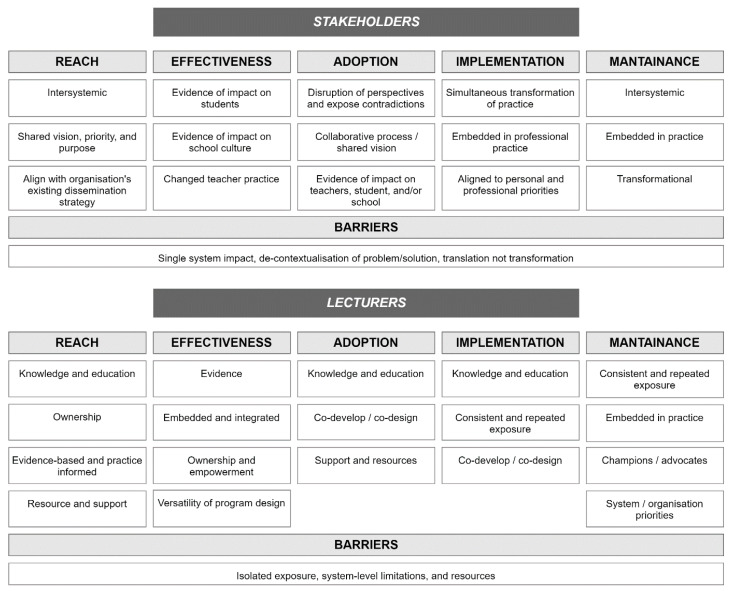
Major themes emerging from stakeholder and lecturer interviews.

Qualitative results from interviews and focus groups with each participant level reported relevant RE-AIM dimensions separately. An overview of the major themes emerging from interviews with stakeholders and lecturers are summarised in Figure 4.

#### 3.3.1. Reach

Three major themes emerged from the stakeholder interviews about enhancing program reach. The first was the need for “inter-systemic” program dissemination. This includes identifying specific “levers” across the multiple systems that may enact change, or conversely, hold current practice in place. Stakeholders unanimously reported that “learning should be across multiple systems” and we need to “broaden our view” of what systems need to be involved.

“To transform practice, you need to work with all levers across multiple systems, the broader the dissemination the greater the likelihood of change.”

(Stakeholder 2)

The second theme was to align or create a “shared vision” with the relevant stakeholders across these systems to generate early and “genuine buy-in”.

“We need to identify a shared problem, and importantly identify this program as the shared solution to the problem. This generates buy-in right from the start.”

(Stakeholder 1)

Thirdly, stakeholders highlighted numerous existing channels for dissemination within the faculty. For example, faculty-wide bulletins and newsletter, whole school meetings, site director meetings, course level days, and major course reviews were highlighted as potential avenues to “get the program on the whole-of-school agenda”.

Five major *reach*-related themes emerged from the lecturer interviews. Knowledge and education were reported as being enablers of *reach*. This was regarding knowledge transmission to both intervention deliverers and intervention receivers. For example, one lecturer reported:

“The pre-service teachers really understood that these were such important skills for all teachers to learn–for classroom management, transitions, and overall effective teaching. They felt that it would help them become better in the classroom. Getting this information out there early and more widely is essential.”

(Lecturer 5)

Synergistic with stakeholders, ownership was also a prominent theme in lecturers’ discussion of the reach of the program. This related to empowering the intervention deliverers, involving them in co-design and giving them ownership of program content, design and delivery.

“I actually felt empowered by it [co-design] as I was gaining knowledge and building a resource bank—this meant I could disseminate the messaging more broadly, more widely and more consistently.”

(Lecturer 1)

Another facilitator to *reach* was that the program was “evidence-based” and practice informed, enabling transferability to practice.

“The results speak for themselves. This is its selling point and how you will get it out there. It’s based on such sound evidence and then when you take this toolbox [active teaching strategies] and experiment first-hand with them, it is transferred immediately.”

(Lecturer 2)

Sufficient provision of support and resources, coupled with flexibility to adapt the program, also emerged as a dominant theme.

“I was able to include all the key concepts and resources in each session but as it is a very versatile program; I could adapt it as needed to reach the different cohorts.”

(Lecturer 2)

Lecturers suggested that if the program could be prioritised at an organisational level and prescribed in policy and curriculum documents (e.g., embedded in unit learning outcomes teacher standards and registration), it may enhance the reach further.

“Exposure to other areas of the curriculum across the education faculties is essential, it needs to be incorporated into all subject areas within the universities.”

(Lecturer 3)

#### 3.3.2. Effectiveness

Three major effectiveness themes emerged from stakeholder data. All three were associated with impact. Stakeholders collectively agreed that evidence of program impact across three separate, but interrelated, levels would be the ultimate measure of effectiveness. This included change in teacher practice, a change in school culture, resulting in the “ultimate indication of program effectiveness”, improved student educational outcomes.

“All other measures of effectiveness are really just proxies. What we really need to show is transformed practice at a school level to improve student outcomes.”

(Stakeholder 1)

“The health, wellbeing and educational outcomes of a student is the ultimate indicator of impact.”

(Stakeholder 3)

Four major themes regarding effectiveness emerged from the lecturer interviews. The lecturers shared that the “effects of the program were immediate” and that the practical and experiential learning provided the pre-service teachers with “real evidence”, and subsequently significantly enhanced their learning.

“The pre-service teachers were not only learning about these strategies but they were experiencing them and experimenting with them. It was this practical and experiential learning that consolidated the theory.”

(Lecturer 4)

Another major theme positively influencing effectiveness was the “embedded nature of the program”. Key content was embedded in each seminar and lecture across all weeks of the unit, as well as being integrated into curriculum documentation and assessment tasks. In addition, lecturers reported that they “actively modelled”, discussed and educated the pre-service teachers around the key aspects of the program in every session. Further, they provided opportunity for the pre-service teacher to experience receiving the content (as a student) and delivering the content (peer micro-teaching).

“I think the pre-service teachers need to see how it works in practice. For this to become a real prospect for them they need to see it, be part of it and practice it. Without that integrated or embedded practice it may be hard for them to translate into their teaching when they are graduates. I think that is one of the best aspects of this program—its embedded across all aspect of the unit, lectures, seminars and assessment.”

(Lecturer 4)

Lecturers reported that their increased levels of ownership and empowerment as a consequence of the co-design process and resultant growth in program knowledge, confidence, and passion positively influenced program effectiveness.

“I am a practising primary school teacher as well as a lecturer, so I was able to contribute a real-life perspective and make this applied. It was like connecting the research to practice and gave me real ownership as I was bringing in real life examples from my teaching.”

(Lecturer 5)

The lecturers also reported that there was significant growth in the pre-service teachers’ confidence and motivation as the program progressed. Moreover, the lecturers perceived that the pre-service teachers enjoyed, were satisfied, and had a positive experience with the program, which subsequently enhanced effectiveness.

“From a lecturer perspective it was observing their [pre-service teachers’] passion and drive grow over the unit; to be active teachers. They realised that there is no other way; this is the only way. For the pre-service teachers, it was the belief in themselves, realising that they can now do it with the skills that they have. They feel equipped with their new ‘teacher toolbox’ to be active classroom teachers.”

(Lecturer 2)

Lecturers suggested that limited exposure of the program, both across other units in the course, as well as other years of the degree, may be a barrier to effectiveness.

“The lack of consistent exposure and dose of active breaks and active teaching across their course will be a challenge. This is the only unit across the entire degree that they are exposed to active teaching the lack of exposure may dilute the message. Also being in first year, they have such a long time before they are out teaching that they may forget the message.”

(Lecturer 1)

Via focus group discussions, pre-service teachers (*n* = 30/30) unanimously reported positive perceptions of the program. Although a few (*n* = 5/30) shared feelings of apprehension at the beginning, many expressed that the program effectively equipped them with skills, strategies, and knowledge, resulting in increased confidence.

“Although I was nervous that I would not have the skills and knowledge to be able to do so, as the unit progressed the excitement remained but the nerves went away as I was provided with many resources and examples of how to conduct physical education classes and involve physical activity into my classroom which made me feel a lot more confident.”

(Pre-service teacher 1)

Many of the pre-service teachers (*n* = 21/30) reflected on the growth in their knowledge and understanding of the importance of integrating physical activity in their teaching.

“My views, feelings and values that I possess surrounding the importance of physical activity levels have been reinforced and strengthened throughout. It has been extremely useful to learn ways in which to incorporate more activity into the generalist classroom. I have a significantly greater understanding of the value of active breaks and the integrated curriculum. I feel a lot more confident to deliver appropriate, active, fun lessons.”

(Pre-service teacher 11)

Most of the pre-service teachers (*n* = 22/30) expressed that the program effectively provided a transferable skillset, viewed as a “value-add to their generalist classroom teacher toolkit”.

“There are so many strategies that I have learnt from this program that I am going to include in my teaching, like including movement-based activities into normal lessons, which was not something that I would have thought about doing. I never realised that generalist teachers could do that sort of thing in their classroom, or even that they needed it. All these strategies that I have learnt are going to improve my teaching in many ways.”

(Pre-service teacher 10)

#### 3.3.3. Adoption

Stakeholder interviews revealed three major adoption themes. Firstly, stakeholders suggested that there needed to be a “disruption of perspectives” and an “exposure of contradictions”. Primarily in unpacking the shared purpose, vision and priorities of pre-service teacher education more broadly among academics, and respectfully but critically highlight how current practice contributes to this purpose, or contradicts it.

“Our primary purpose, our work here is to teach people, not just to teach content. We need to make the connection between teacher practice and student outcomes, regardless of content. By understanding this sole purpose, we, the collective we, may begin to see the disconnect between what we to do and what we actually need to do. However, we should not only expose this contradiction but provide a solution.”

(Stakeholder 4)

“We need to understand what holds things [current practice] in place. Once we understand this we can start to shift or transform this.”

(Stakeholder 1)

The second theme that emerged as a facilitator to program *adoption* regarded utilising an “authentic collaborative process to create a shared vision”.

“It [Transform-Ed!] should be based on shared ideas and true collaboration—both researcher and lecturers need to be willing to share, learn and change.”

(Stakeholder 3)

The third theme was ‘evidence of impact’. Specifically, stakeholders shared that the program had the capacity and potential to transform initial education, teacher practice and student outcomes simultaneously.

“The beauty of this program is that it situates in the middle of the conversation, the high-end measure here which is student learning. If we can show improved student outcomes, you know that you are changing teacher practice. With this evidence you will have more people willing to adopt.”

(Stakeholder 1)

Three themes emerged from the lecturer interviews. Again, most prevalent was knowledge and education. The lecturers conveyed that the more they learned about and experienced the program, and the more they observed the results, the more willing and passionate they were to adopt it.

“The more I learn about this program and the more I see the impacts of it, the more convinced I am to use it in my teaching.”

(Lecturer 1)

The lecturers reported that the co-design/co-develop aspect of the program enhanced feelings of ownership and empowerment. They reported on the idea of personal “buy-in” and “passion” for the program. In addition, the lecturers stated that the co-design aspect ensured that the program was practice informed. As such, the lecturers used their experience as teachers to feed into the evidence-based program.

“Researching additional activities and bringing examples from my own teaching made me feel truly part of this. I could then also advocate for these activities as I knew they really worked in practice. I had ownership and could authentically add value to what was already there.”

(Lecturer 4)

The lecturers reported that although they felt the support and resources provided were sufficient, an increased amount and diversity of resources, especially around active breaks and active lessons, may have led to greater levels of adoption.

#### 3.3.4. Implementation

The major implementation themes emerging from stakeholder interviews included the simultaneous transformation of practice, being embedded in professional practice and alignment to personal and professional priorities. Regarding the simultaneous transformation of practice, the stakeholders referred to how a change in pre-service teacher practice required concurrent changes at the school level. They made mention of “true implementation impact”, suggesting that the “life” that a practice, tool, resource or artefact takes on in a school is the real indictor of successful program implementation.

“Making the tool or resource [available] is not enough, you need to implement it and embed it in practice, to ultimately transform practice. This is when the artefact takes on a life of its own. It is then that you can measure this change in practice. And that is your impact.”

(Stakeholder 1)

Similarly, implementing the program into professional practice emerged as a prominent enabler. Stakeholders made mention of the need for “clearer links, connections and pathways to professional practice” to enable broader and more effective implementation. Consistent messaging from placement supervisors, coordinators and placement setting staff, to ensure pre-service teachers feel well equipped and supported to embed the practices into their teaching was also perceived as important. Stakeholders again reported on the need for “true collaboration with a broad range of potential deliverers” to understand priorities and practices and, subsequently, create a shared vision for program implementation.

“To take this to other areas of the curriculum and bring on board more people to implement the strategies, first you need to understand why they do what they do, why they don’t already teach in this way, and then you need to make clear links and benefits of this program to their practice. You will need to be open to learning and shared understanding, just as they will be—this is true collaboration.”

(Stakeholder 2)

Three major themes emerged from lecturer interviews. Consistent with previous domains, knowledge and education again emerged as dominant. Lecturers conveyed that the more they were educated about the program the more empowered, confident and motivated they felt to implement the program. Lecturers also reported that their previous exposure and experience in delivering the active breaks and lessons (e.g., via the feasibility study) was invaluable, and again contributed to increased feelings of confidence.

“When I delivered it for the second time, I then knew exactly what and where the breaks and activities were. I was much more confident and well versed the second time around. I actually think seeking out my own active breaks gave me more ownership and confidence in the unit. I knew exactly what I had to do and when I needed to do it.”

(Lecturer 3)

The lecturers also perceived that knowledge and education were powerful “change agents” in regard to pre-service teachers’ attitude, confidence, and willingness to implement the Transform-Ed! key messages in their current (peer micro-teaching) and future (school) teaching.

“A lot of pre-service teachers came in saying they were not skilled enough, not confident enough to do the unit. They realised quickly that these skills and strategies were accessible to all. They quickly understood the importance of integrating physical activity into their teaching, and how transferable they are to teach any content in the curriculum.”

(Lecturer 1)

A theme that emerged as both a facilitator and barrier to *implementation* was “consistent exposure”. Congruent with their adherence checklist data, the lecturers conveyed that as the content was formally embedded and integrated across every aspect of the targeted unit (i.e., in lectures, seminars, assessment, curriculum planners, discussion board posts, experiential learning experience) it allowed for consistent and repeated exposure of program messaging across that unit. Of note was the practical application of the messages, both in regard to lecturer modelling of practices, as well as pre-service teacher experiential learning. However, unanimously, the lecturers reported that exposure of the program in one unit only is not enough to elicit long term change in teacher practice, school culture and student outcomes.

“I think we need more ‘champions’–more people demonstrating these pedagogies–so that the pre-service teachers have constant exposure to it. More units across first year and also the program spread across more years of the course.”

(Lecturer 2)

As with adoption, another influential implementation related theme was the co-design/co-develop aspect of the program. Lecturers conveyed that they felt ‘empowered’ and shared feelings of ‘program ownership’ which facilitated implementation.

“By choice I invested in a range of resources for lecture active breaks. This was not essential and could be avoided, but I was empowered to provide as many examples as I could, so I happily sourced out these extra activities and examples.”

(Lecturer 1)

Pre-service teachers unanimously (*n* = 30/30) shared that the program had a positive influence on their perceived ability to implement diverse teaching strategies. This particularly regarded increased knowledge gained, the practical and transferable nature of the strategies and the “value now placed on classroom-based activity”. What follows is just an example of the multiple comments pre-service teachers made in relation to the perceived value of the program:

“With the knowledge I have received from this unit I now have lots of ideas and activities that I can incorporate. I will aim to integrate these things into the curriculum to enhance the children’s learning processes and reduces the likelihood of the students becoming distracted, especially if they are being active and moving around rather than being sedentary.”

(Pre-service teacher 21)

#### 3.3.5. Maintenance

The major themes to emerge from stakeholder interviews regarding *maintenance* included the need for an inter-systemic approach, for the program to be embedded in professional practice and for the program to provide a “transformation of practice rather than just translation into practice”. Stakeholders also shared the importance of understanding what holds current practice in place, what prevents change.

“There is a need to have all the key players or levers across multiple systems, sitting around the one table at the one time. This includes people like the timetablers, resource and facility managers. These people are often left out but are actually critical to the logistics of making or preventing change.”

(Stakeholder 1)

Stakeholders also reinforced the need to “create clear pathways into professional practice” and then measure the impact of the program on teacher practice and student outcomes. The final theme that emerged was the “need for transformation, rather than just translation”.

“Numerous programs can be translated from research to practice or from setting to setting. But for a program to really stand the test of time, it needs to be transformational. To enact change in and on the human system.”

(Stakeholder 1)

Following the implementation trial, the program has been sustained for two additional trimesters, by all lecturers at the three campuses. Further adaptations have been made by the lecturers to enhance contextual fit. This has largely included greater exposure to active strategies via modelling, the provision of more resources and further opportunities for peer teaching and greater emphasis and priority in assessment tasks. Moreover, lecturers were able to adapt the program quite significantly to successfully deliver in an intensive format in Term 3, 2019, and an online format due to the impact of Coronavirus (COVID-19) restrictions in Term 1, 2020.

“I was able to include concepts even in the variation in delivery due to COVID-19. For the online lectures we were quite creative by providing videos to demonstrate the active academic sessions which the preservice teachers really enjoyed”.

(Lecturer 3)

Four major themes related to perceived facilitators of maintenance, emerged from the lecturer interviews. Again, the idea of consistent and repeated exposure of messaging across the unit was prevalent.

“They [pre-service teachers] need to see the consistent and repeated exposure of these practices. Without this, by fourth year it will be diluted. If they can see it modelled and have experimentation with teaching across their whole course and in professional practice. This reaffirms what they are learning and will help them embed it into their teaching practice.”

(Lecturer 2)

Similarly, the program being “embedded in practice” was a perceived facilitator of program *maintenance*. Lecturers reported that as the content was embedded in lecture and practical curriculum and modelling, in set peer teaching opportunities and experiential learning, “the program just become part of [their own] regular teaching practice”. However, lecturers also shared that if the program were formally embedded into teacher professional practice, it would have a more sustained impact on pre-service teachers.

“It [Transform-Ed!] needs to be built into the professional practice experience, and there needs to be more support. The more they experience it in practice, the more these concepts will be consolidated and become part of their regular practice.”

(Lecturer 2)

To further enhance program maintenance, lecturers suggested incorporating ‘*program champions*’. They reported that early adopters (i.e., lecturers) and undergraduate teachers would be well placed to advocate more broadly for the program.

“We could also rely on the pre-service teachers—we have given them some foundational knowledge and strategies to deliver curricula actively and they have had such a positive experience. If they question other lecturers as to why they are not seeing these strategies in their lectures or seminars—the demand may ignite interest in more lecturers.”

(Lecturer 4)

The lecturers suggested that there needs to be system or organisational level changes for the program to be expanded, prioritised and sustained. They suggested that the program needs to be embedded across more units and more years of the course, embedded within professional placement experiences in schools, and prioritised in Graduate Teacher Standards.

“There needs to be a system level approach to this. Modelled from the top down and bottom up—if we can get it into all aspects of teaching and learning, as research, then I think we will have a chance to really impact teaching.”

(Lecturer 2)

The majority of the pre-service teachers (*n* = 25/30) reflected that the skills and strategies learned across the unit, would be very much part of their future teaching, and that this program has “shaped” and “informed” the type of teacher they wanted to become.

“This has given me a broader understanding of physical activity and how I can incorporate greater activity into my classes when I become a teacher. I know how important it is to get kids active and I will aim to do this in all aspects of my teaching.”

(Pre-service teacher 2)

Not only did the teachers report that they would incorporate these strategies into their own teaching, but many (*n* = 18/30) suggested that they would “advocate for change” or be “champions of active teaching” when out in schools.

“My feelings towards the importance of physical activity in school have definitely developed and I have gained a wider knowledge on it. That strategies that I have learnt in this unit will be reflected in my teaching when I become a teacher and will shared with all the other staff at my school.”

(Pre-service teacher 7)

## 4. Discussion

This study is the first to use implementation theory to understand the potential of pre-service teacher training to support school-based physical activity interventions in real-world school systems. The primary purpose of this research was to investigate the reach, effectiveness, adoption, adaption, implementation and maintenance of Transform-Ed! across the first year of an undergraduate teacher education unit, using the RE-AIM framework. Overwhelmingly, the Transform-Ed! program was received positively by key stakeholders, lecturers and pre-service teachers alike. The program improved pre-service teachers’ perceived competence, confidence, and willingness to integrate active pedagogies into current and future teaching practice, and their emerging identities as teachers. Indeed, the impact of the program on pre-service teachers’ perceptions was more substantial than what we found in the pilot study, particularly in regards to their willingness to implement the strategies [36]. The lecturers reported that the program was very engaging and enhanced their understanding of and attitudes toward active learning models. Adherence to the program was high during the 12-week trial and lecturers also adapted and maintained the program after the trial.

Progress in school-based physical activity interventions has been potentially hampered by a focus on individual level, short-term effectiveness trials targeting currently practicing teachers. To address this, the current research targeted pre-service teacher education in an upstream approach which comprehensively used all five RE-AIM framework domains [22] to design, implement and evaluate Transform-Ed!. The rigorous consideration of the RE-AIM dimensions illuminated key learnings and broader issues to consider for future iterations of the program. Four key themes emerged spanning multiple dimensions and participant levels: (i) the importance of an “inter-systemic approach”; (ii) a “co-design” approach; (iii) “embedding the program into professional practice”; and (iv) “evidence of impact” on teacher practice.

The inter-systemic approach refers to organisational level involvement. The broader the scope, or reach, of the system, the more actors would be influencing the ‘human system’, lending a greater likelihood of effect. This is congruent with ‘action theory’, which enables systematic learning of what works and what does not work at the organisational level [50]. Glasgow et al. [51] emphasised the importance of engaging key stakeholders and necessary decision-makers early and often to determine priorities, justify the need for intervention and sustain implementation [51]. In the present study, early and continuous involvement of stakeholders situated across the education systems was already a key feature. However, beyond this, key stakeholders suggested broader dissemination of knowledge across multiple systems (e.g., policy makers, assessment and curriculum authorities, education providers, placement schools, facility managers and timetablers) to allow participants to contextualise and subsequently prioritise the program, facilitating a shared vision. Existing networks and communication channels (e.g., weekly bulletins and whole-of-school forums) were recommended to reach more participants via contextually appropriate, familiar, and relevant channels. This is congruent with Harden et al.’s (2018) recommendations to enhance public health impact [52] by leveraging off existing systems in the organisational setting to enhance local relevance [51].

Co-design emerged as an important feature across the *adoption*, *implementation,* and *effectiveness* dimensions. In particular, co-design here refers to processes that create a shared vision among participants, which has been viewed as effective in the development of school-based physical activity frameworks [39]. Co-design was an important feature built into the present study, as it was framed by participatory action research through all stages of the trial. Here, the participatory nature specifically referred to active involvement and capacity-building with key stakeholders and lecturers to increase ownership of and belief in the program [40]. Key stakeholders (e.g., course directors and unit chairs) assisted in the co-creation of the Transform-Ed! curriculum and aligned it with the priorities, structure, objectives and desired outcomes of the School of Education. This engagement via a consultative process ensured the program was aligned to the relevant needs of users, which has been shown to improve implementation [53], but also enhanced authentic collaboration and ownership at crucial participant levels, subsequently increasing adoption, adaption and delivery of the program [54]. Therefore, it is recommended that broader levels of organisational support should be pursued to adopt and deliver Transform-Ed!, in a bid to expand and disseminate the program across multiple units and years of the course [52].

With the lecturers, the intention of participatory action research was that they were active contributors to the research, and had increased control over the design and delivery of the Transform-Ed! program [40]. A critical aspect of participatory action research in the current research was the collective, self-reflective inquiry that researchers and participants undertook, so they could understand and improve on their own practices [40]. This is congruent with communities of practice, where the value lies in the depth of participants’ reflection and inquiry, and how co-created knowledge is put to action in the local setting to improve performance and practice [55]. Here, the lecturers reflected on the empowering nature of the research process, which led to increased buy-in. Importantly, the reflective process of participatory action research was also directly linked to action, leading to transformation of practice [56], which was evidenced by the lectures adapting and improving their own practice. The program design elements that stimulated creation of a shared vision and program ownership were considered highly valuable by participants.

The third major theme was the importance of embedding Transform-Ed! into professional practice, which emerged across the effectiveness, implementation, and maintenance dimensions. This means that the program needs to be fully rooted in the teaching practice, providing easy transferability and relevance of the physically active pedagogical skills. This is congruent with recommendations by the Teacher Education Ministerial Advisory Group, suggesting that initial teacher education should be a mutually reinforcing experience of higher education and professional learning and practice [31]. All participants articulated that clear pathways to school-based teaching experience and an embedded professional practice placement would further consolidate the key Transform-Ed! messages and provide context, relevance, and meaning to the program content. Previous research has highlighted the importance of professional practice on teacher preparation, as it provides real opportunities for pre-service teachers to integrate theory and practice [46]. Therefore, academic teacher education should be integrated with practice in schools so that it becomes fused with real world usage [31]. Thus, it will be critical to provide clear pathways to more comprehensively embed the Transform-Ed! program into professional practice in future research.

The final of the four major themes was the importance of evidence of impact, in regard to both adoption and effectiveness of the program. Evidence of impact refers to both the evidence base that the program is built on (e.g., Transform-Us!), as well as evidence that the Transform-Ed! program works. Key findings from the Teacher Education Ministerial Advisory Group [31] reveal that not all initial teacher education programs equip graduates with the content knowledge, evidence-based teaching strategies and skills they need for teaching practice [31]. As such, Transform-Ed! may address this inadequacy in existing initial teacher education provisions by providing the evidence to underpin key elements of initial teacher education, from the design and delivery of programs to the practices taught within programs. However, participants readily agreed that the ultimate measure of program effectiveness would be evidence of impact at the primary school student level, which was beyond the scope of the present study. Research has demonstrated that active pedagogy can benefit children’s cognition, classroom behaviour and academic behaviour [12], so this will need to be a priority in future Transform-Ed! research.

Regarding evidence of impact, the main limitation of this trial was that it did not measure the impact of Transform-Ed! on primary school children’s physical activity, sedentary behaviour, or engagement with their learning or academic outcomes. As mentioned, future research is needed to examine the real-world impact of Transform-Ed!. It was readily noted by key stakeholders that the ultimate measure of effectiveness would be improved student outcomes, as this would be simultaneously indicative of changed teacher practice and changed school culture [57]. It has been argued that truly knowing the nature and magnitude of your impact as a teacher requires evidence of the effects of practice on student outcomes [57]. Indeed, the ultimate goal of school interventions is to advance outcomes for students, so direct measures of this are essential [57]. Although pre-service teachers in the present study reported enhanced confidence and perceived competence to use active pedagogy in the classroom, future research will need to investigate the impact of Transform-Ed! on their capacity to increase primary school children’s physical activity and academic-related outcomes. Initially, this could be measured during student placements, which were not a feature of the unit in the present study, then again later when they gain employment as in-service teachers in schools.

## 5. Conclusions

The results of this implementation trial of Transform-Ed! reinforced decisions about its design, and inspired enhancement of these features in future scaled-up version of the program. For instance, heavy involvement at the organisational level and emphasis on a process of co-design should be retained and taken even further. Participants reported that the program needs to be embedded more comprehensively in professional practice, for example, by expanding the program across units and years of the teaching degree and via professional practice teaching placements. Perhaps, most importantly, evidence of impact would need to be scaffolded in future research, primarily by determining the effectiveness of the program on improving primary school student activity levels and engagement in learning and academic outcomes, and also investigating the potential for flow-on effects of the program to other teachers in placement schools. According to the present study, targeting pre-service teacher education appears to be a promising avenue to enhance the capability of Australian teachers and, in time, hopefully transform the learning experience and outcomes of students.

## Figures and Tables

**Table 1:** Lecturer-to-pre-service teacher implementation aspects.

Strategy	Elaboration	Strategy
Active academic lessons	Normal planned lessons, where the delivery method rather than the content is changed.	Modelled active academic teaching strategies in lectures and practical seminars.
		Integrated pedagogical theory (e.g., embodied pedagogy) and practice (e.g., skills, strategies, organizational, and managerial concepts) to facilitate active academic lessons.
		Provided resources for active academic lessons.
		Provided opportunity for pre-service teachers to practice skills, strategies, organizational and managerial concepts required to teach active academic lessons.
		Provide opportunity for self, peer, and lecturer feedback on pre-service teachers’ active academic micro-teaching.
Active breaks from sitting	During extended teaching blocks, short active breaks were used interrupt prolonged periods of sitting.	Modelled active beaks in lectures and seminars.
		Integrated pedagogical theory and practice (skills, strategies, organizational and managerial concepts) to facilitate active breaks.
		Provided active break resources.
		Provided opportunity for pre-service teachers to practice skills, strategies, organizational, and managerial concepts required to break sitting time.
		Provided opportunity for self, peer, and lecturer feedback on pre-service teachers’ active break micro-teaching.
Transform-Ed! Health Lesson Curriculum Content	Class lessons, which aim to build skills and increase knowledge about the importance of being active and sitting less.	Provided information around the importance of adequate physical activity.
		Provided resources for future teaching around the importance of physical activity.
		Provided opportunity for pre-service teachers to practice skills, strategies, organizational, and managerial concepts required to deliver physical activity related content in micro-teaching.
		Provided opportunity for self, peer, and lecturer feedback around their physical activity related content micro-teaching.
Active environments/promoting activity during recess and lunchtime	Signage/posters, equipment/facilities and teacher encouragement promoting physical activity at recess and lunchtime.	Delivered seminar/lecture focused on playground-based activities that facilitate physical activity at recess/lunchtime.
		Provided resources for playground-based activities.
		Provided opportunity for pre-service teachers to practice skills, strategies, organizational and managerial concepts required to facilitate playground-based activities, in micro-teaching.
		Provided opportunity for self, peer and lecturer feedback around their playground activities micro-teaching.
Engaging families	Newsletters and activities provided for parents and children to engage with, to reinforce the importance of children being active and sitting less.	Delivered seminar/lecture on active homework strategies that engage families and educate around the importance of increasing Physical activity and decreasing sitting time at home.
		Provided information around the importance of engaging families and the community when addressing physical activity behaviour (e.g., ecological model).
		Provided active homework resources.
		Provided opportunity for active homework activities, micro-teaching.
		Provided opportunity for self, peer, and lecturer feedback around their active homework tasks.

**Table 2:** The design, implementation and evaluation of Transform-Ed! as guided by the RE-AIM (reach, effectiveness, adoption, implementation, and maintenance) framework.

RE-AIM Dimension/Item	Participant Level	Evaluation
Reach		
Program dissemination	Stakeholders	*The number, proportion and representativeness of stakeholders willing to participate in the study* *Quantitative* Number of senior academics identified and sent email invitations to participate in presentations and interviews Number of senior academics attending presentations and interviews Characteristics of participants: roles and responsibilities of stakeholders *Qualitative* Post-program interviews to understand barriers and facilitators to reach and/or recruitment
	Lecturers	*The number, proportion and representativeness of lecturers willing to participate* *Quantitative* Number of lecturers/tutors/sessional staff sent email invitations to participate in the study *Qualitative* Post-program interviews to understand barriers and facilitators to reach and/or recruitment
	Pre-service teachers	*The number, proportion and representativeness of pre-service teachers willing to participate* *Quantitative* Number pre-service teachers enrolled in the first year Bachelor of Education (primary) degree Number of pre-service teachers emailed program advertisement, invitation, plain language statement and consent to participate
Pre-implementation Adaptation	Stakeholders	*The number, proportion and representativeness of stakeholders who were willing to participate in pre-program discussions* *Quantitative* Number participating in discussion, # participating in post-program interviews
Pre-implementation Adaptation	Lecturers	*The number, proportion and representativeness of lecturers willing to participate in pre-program discussions and curriculum co-design* *Quantitative* Number of lecturers involved in curriculum re-development discussions and co-design (moving from feasibility to implementation) Number of lecturers involved in interviews *Qualitative* Discussions to reflect on feasibility/pilot study and integrate learnings
Effectiveness		
	Lectures	*Measures of primary outcome* *Qualitative (interviews)* • Perceived competence, confidence, and willingness to use active strategies in current and future teaching • Perceived impact of intervention on pre-service teachers
	Pre-service teachers	*Measures of primary outcome* *Quantitative (pre/post self-report surveys)* • changes in competence/confidence and willingness to integrate active pedagogies into current and future teaching *Qualitative (post-program focus group discussions)* • Impact of Transform-Ed! on their emerging identity as teachers
Adoption		
	Stakeholders	*The number, proportion, and representativeness of settings and intervention deliverers; variation of adoption across settings and deliverers* *Quantitative* Percent of staff invited that participated *Qualitative (post-program interviews)* Specific positions/roles represented Characteristics of settings participating Barriers and facilitators to adoption at setting level
	Lecturers	*The number, proportion, and representativeness of settings and intervention deliverers; variation of adoption across settings and deliverers* *Quantitative* Proportion of those receiving compared with # delivering the program Characteristics and qualification of staff delivering the program Settings the intervention is delivered in *Qualitative (post-program interviews)* Interviews to understand staff participation and barriers and facilitators to program adoption
	Pre-service teachers	*Quantitative* Proportion of those involved in Transform-Ed! peer micro-teaching compared with # enrolled in unit
Implementation		
	Stakeholders	*Qualitative (post-program interviews)* Interviews to understand barriers and facilitators to program implementation
	Lecturers	*The intervention deliverers’ fidelity to the intervention, including consistency of delivery as intended and the time and cost of the intervention; adaptations made to interventions and implementation strategies* *Qualitative (post-program interviews)* Interviews to understand barriers and facilitators to implementation Self-report adherence checklist of key Transform-Ed! elements–delivery lecturer to pre-service teacher
	Pre-service teachers	*Qualitative aspects (post-program focus groups)* Barriers and facilitators to implementation
Maintenance		
	Stakeholders	*The extent to which the program could become institutionalized or part of the routine practices* *Qualitative (post-program interviews)* Interviews to understand barriers and facilitators to maintenance
	Lecturers	*The extent to which the program has or could become institutionalized or part of the routine practices; the long-term effects of the program on teaching practices after the intervention is completed* *Qualitative (post-program interviews)* Barriers and facilitators to maintenance
	Pre-service teachers	*The perceived long-term effect of the program on current and future teaching practices after the intervention is completed* *Qualitative (post-program focus groups)* Emerging identity as teachers and whether active pedagogy is likely to be prioritized in professional practice future practice

**Table 3:** Summary statistics of lecturer adherence checklist responses by domain.

	Mean	SD
Active academic lessons	4.24	0.46
Active breaks from sitting	4.40	0.20
Health-related content	4.75	0.18
Active environment	3.55	0.78
Engaging families	3.74	0.82

Note: the possible score for each domain ranged between 1 (poor adherence) and 5 (excellent adherence). Standard Deviation (SD).

## Supplementary Materials

The following are available online at https://www.mdpi.com/2227-9067/7/11/207/s1. Supplementary File S1: Measuring the Use of the RE-AIM Model Dimension Items Checklist; Supplementary File S2: Lecturer-to-pre-service teacher implementation aspects; Supplementary File S3: Stakeholder interview guide; Supplementary File S4: Lecturer interview guide; Supplementary File S5: Results from linear mixed models used to assess the intervention effects on factors associated with successful implementation of active pedagogic strategies in pre-service teachers.
