# Supplementary material for: Embedding Active Pedagogies within Pre-Service Teacher Education: Implementation Considerations and Recommendations
Source: Children (Basel). 2020 Nov 2;7(11):207. doi: 10.3390/children7110207 (PMC7692750; doi:10.3390/children7110207)
Supplement: Supplementary file 1 [file children-07-00207-s001.zip › Supplementary files/Supplementary file 1.pdf]

Measuring the Use of the RE-AIM Model Dimension Items
Checklist

The Implementation Science Team at the National Cancer Institute (NCI) Division of Cancer Control and Population Sciences (DCCPS), in partnership with other key leaders and RE-AIM authors, developed and piloted a 2 page instrument to aid those interested in applying RE-AIM (Reach, Effectiveness, Adoption, Implementation, and Maintenance) to their activities. For each dimension, a list of items which indicate exemplar use of RE-AIM is provided.

This instrument was designed as part of project to review grant proposals for the extent to which they have used RE-AIM and different elements of the framework in their grant applications (manuscript forthcoming). It could easily be adapted for use in planning or reviewing programs or policies, or in drafting grants or journal articles and other reports using the RE-AIM framework.

This coding sheet is an expanded and updated version of earlier coding forms that have been used in reviewing the health promotion literature, but is designed specifically for those wishing to employ RE-AIM.

**Study Topic Area:**	**Study Setting:**
**Dimensions/Items**	**Included?** (Yes, No, Yes-Inappropriate Use, N/A)
**Reach**	
Exclusion Criteria (% excluded or characteristics)	
Percent individuals who participate, based on valid denominator (not of volunteers who indicate interest)	
Characteristics of participants compared to non-participants or to target population	
Use of qualitative methods to understand reach and/or recruitment	
**Effectiveness**	
Measure of primary outcome with or w/o comparison to a public health goal (e.g. HP 2020 goals, exercise 30 min/day; eat 5 Fruits &Veggies)	
Measure of broader outcomes (e.g., other outcomes, measure of QoL or potential negative outcome) or use of multiple criteria	
Measure of robustness across subgroups (e.g. moderation analyses)	
Measure of short-term attrition (%) and differential rates by patient characteristics or treatment condition	
Use of qualitative methods/data to understand outcomes	
**Adoption – Setting Level**	
Setting Exclusions (% or reasons)	
Percent of settings approached that participate (valid denominator)	
Characteristics of settings participating (both comparison and intervention) compared to either: non participants **or** some relevant resource data	

Adoption – Setting Level (continued)	
Use of qualitative methods to understand adoption at setting level	
**Adoption – Staff Level**	
Staff Exclusions (% or reasons)	
Percent of staff invited that participate	
Characteristics of staff participants vs. non participating staff or typical staff	
Use of qualitative methods to understand staff participation	
**Implementation**	
Percent of perfect delivery or calls completed, etc. (e.g., adherence or consistency)	
Adaptations made to intervention during study	
Cost of intervention (time or money)	
Consistency of implementation across staff/time/settings/subgroups (not about differential outcomes, but process)	
Use of qualitative methods to understand implementation	
**Maintenance – Individual Level**	
Measure of primary outcome (with or w/o comparison to a public health goal) at ≥ 6mo follow-up after final intervention contact	
Measure of broader outcomes or use of multiple criteria at follow-up (e.g., measure of QoL or potential negative outcome) at follow-up	
Robustness data - something about subgroup effects over the long-term	
Measure of long-term attrition (%) and differential rates by patient characteristics or treatment condition	
Use of qualitative methods data to understand long -term effects	
**Maintenance- Setting Level**	
If program is still ongoing at ≥ 6 month post study funding	
If and how program was adapted long-term (which elements retained AFTER program completed)	
Some measure/discussion of alignment to organization mission or sustainability of business model	
Use of qualitative methods data to understand setting level institutionalization	
