# Supplementary material for: Embedding Active Pedagogies within Pre-Service Teacher Education: Implementation Considerations and Recommendations
Source: Children (Basel). 2020 Nov 2;7(11):207. doi: 10.3390/children7110207 (PMC7692750; doi:10.3390/children7110207)
Supplement: Supplementary file 1 [file children-07-00207-s001.zip › Supplementary files/Supplementary file_2.docx]

***Supplementary file 2:*** *Lecturer-to-pre-service teacher implementation aspects*

| **Strategy** | **Elaboration** | **Strategy** | **1=not at all true**  **5=very true** |
| --- | --- | --- | --- |
| Active academic lessons | Normal planned lessons, where the delivery method rather than the content is changed. | - Modelled active academic teaching strategies in lectures and practical seminars. | 1, 2, 3, 4, 5 |
|  |  | - Integrated pedagogical theory (e.g., embodied pedagogy) and practice (e.g., skills, strategies, organisational and managerial concepts) to facilitate active academic lessons. | 1, 2, 3, 4, 5 |
|  |  | - Provided resources for active academic lessons. | 1, 2, 3, 4, 5 |
|  |  | - Provided opportunity for pre-service teachers to practice skills, strategies, organisational and managerial concepts required to teach active academic lessons. | 1, 2, 3, 4, 5 |
|  |  | - Provide opportunity for self, peer and lecturer feedback on pre-service teachers active academic micro-teaching. | 1, 2, 3, 4, 5 |
| Active breaks from sitting | - During extended teaching blocks, short active breaks were used interrupt prolonged periods of sitting. | - Modelled active beaks in lectures and seminars. | 1, 2, 3, 4, 5 |
|  |  | - Integrated pedagogical theory and practice (skills, strategies, organisational and managerial concepts) to facilitate active breaks. | 1, 2, 3, 4, 5 |
|  |  | - Provided active break resources. | 1, 2, 3, 4, 5 |
|  |  | - Provided opportunity for pre-service teachers to practice skills, strategies, organisational and managerial concepts required to break sitting time. | 1, 2, 3, 4, 5 |
|  |  | - Provided opportunity for self, peer and lecturer feedback on pre-service teachers’ active break micro-teaching. | 1, 2, 3, 4, 5 |
| Transform-Ed! Health Lesson  Curriculum Content | - Class lessons, which aim to build skills and increase knowledge about the importance of being active and sitting less. | - Provided information around the importance of adequate physical activity. | 1, 2, 3, 4, 5 |
|  |  | - Provided resources for future teaching around the importance of physical activity. | 1, 2, 3, 4, 5 |
|  |  | - Provided opportunity for pre-service teachers to practice skills, strategies, organisational and managerial concepts required to deliver physical activity related content in micro-teaching. | 1, 2, 3, 4, 5 |
|  |  | - Provided opportunity for self, peer and lecturer feedback around their physical activity related content micro-teaching. | 1, 2, 3, 4, 5 |
| Active environments/  Promoting activity during recess and lunchtime | Signage/posters, equipment/facilities and teacher encouragement promoting physical activity at recess and lunchtime. | - Delivered seminar/lecture focused on playground-based activities that facilitate PA at recess/lunchtime. | 1, 2, 3, 4, 5 |
|  |  | - Provided resources for playground-based activities. | 1, 2, 3, 4, 5 |
|  |  | - Provided opportunity for pre-service teachers to practice skills, strategies, organisational and managerial concepts required to facilitate playground-based activities, in micro-teaching. | 1, 2, 3, 4, 5 |
|  |  | - Provided opportunity for self, peer and lecturer feedback around their playground activities micro-teaching. | 1, 2, 3, 4, 5 |
| Engaging families | Newsletters and activities provided for parents and children to engage with, to reinforce the importance of children being active and sitting less. | - Delivered seminar/lecture on active homework strategies that engage families and educate around the importance of increasing PA and decreasing sitting time at home. | 1, 2, 3, 4, 5 |
|  |  | - Provided information around the importance of engaging families and the community when addressing physical activity behaviour (e.g. ecological model). | 1, 2, 3, 4, 5 |
|  |  | - Provided active homework resources | 1, 2, 3, 4, 5 |
|  |  | - Provided opportunity for active homework activities, micro-teaching. | 1, 2, 3, 4, 5 |
|  |  | - Provided opportunity for self, peer and lecturer feedback around their active homework tasks. | 1, 2, 3, 4, 5 |
