# Supplementary material for: Embedding Active Pedagogies within Pre-Service Teacher Education: Implementation Considerations and Recommendations
Source: Children (Basel). 2020 Nov 2;7(11):207. doi: 10.3390/children7110207 (PMC7692750; doi:10.3390/children7110207)
Supplement: Supplementary file 1 [file children-07-00207-s001.zip › Supplementary files/Supplementary file_3.docx]

***Supplementary file 3:*** *Stakeholder interview guide*

| **RE-AIM elements** | **Discussion Prompts** |
| --- | --- |
| Reach | - What could the team have done (or do better) to reach more people?   - Or reach different people in the School of Education?   - If so who and why? |
| Effectiveness | - Are these relevant measures of program effectiveness   - Why/Why not?   - are they aligned to organisation priorities?   - If so how, if not why?   - What can be altered? - What other effectiveness measures would you like to see investigated and/or included?   - Why are these important to explore? |
| Adoption | - What are your perceptions of the training that was offered? - What could have been done differently? Why/how? |
| Implementation | - Many staff self-reported and were observed delivering the intervention with fidelity. Please describe why you think this was?   *The ultimate aim for any behaviour change intervention is sustainability and scalability. The next step for Transform-Ed! is scalability.*   - What do you perceive to be the major barriers and facilitators to the scaled-up implementation of Transform-Ed! within the Bachelor of Education (primary)?   - The School of Education more broadly?   - Across different universities? - What are/could be some suggested solutions to barriers? |
| Maintenance | - What is the likelihood that you would continue to support this intervention? - What would be needed for other stakeholders within the School of Education to find this intervention meaningful? - Can it be sustained? - What is needed for this to occur? - Does this, or how could this, initiative align with current and future policies and priorities? |
