# Supplementary material for: Embedding Active Pedagogies within Pre-Service Teacher Education: Implementation Considerations and Recommendations
Source: Children (Basel). 2020 Nov 2;7(11):207. doi: 10.3390/children7110207 (PMC7692750; doi:10.3390/children7110207)
Supplement: Supplementary file 1 [file children-07-00207-s001.zip › Supplementary files/Supplementary file_4.docx]

***Supplementary file 4:*** *Lecturer interview guide*

| **RE-AIM elements** | **Discussion Prompts** |
| --- | --- |
| Pre-implementation adaption  (planning phase) | - What were your initial perceptions and thoughts of Transform-Ed! - What did you want the intervention to achieve (impact)– personally, for your students (pre-service teachers), more broadly for teacher education - Who did you think should be involved in the delivery and receiving of the Transform-Ed! program? - How did you think it should or could it be delivered and sustained? - *Pre-delivery* - what did you perceive the barriers and facilitators to implementation would be? - Can you share how you modified the program prior to program delivery in 2019   - Evidence of pre-implementation adaptation (moving from feasibility 2018 to implementation 2019)     - Curriculum/weekly planners     - lectures/seminars     - Resources     - Assessments     - Delivery methods     - other |
| Reach | - What could the team have done (or do better) to reach more people? Or to reach different people in the School of Education? If so who, how and why? |
| Effectiveness | - Share your thoughts on whether the Transform-Ed! program was effective at an individual and organisational level. - What other effectiveness measures would you like to see investigated and/or included as part of a teacher education program? Why are these important to explore? - What were the key outcomes of the intervention in regard to both lecturers/pre-service teacher practice? - What do you perceive were the greatest barriers and facilitators to the effectiveness of Transform-Ed!   - User perceptions (lecturers/pre-service teachers)   - Capacity or readiness of lecturers and pre-service teachers   - System/policy level barriers and facilitators (unit level/course level/unit chair level/etc) |
| Adoption | - Can you tell me about your qualifications and experience in regard to teaching and teacher education … what about active pedagogies? - What are your perceptions of the training that was offered? - What could have been done differently? Why/how? - Transform-Ed! was delivered across 3 campuses by 7 different people. What do you think the differences (of adoption) across settings and across staff were and why? - What were the major barriers and facilitators to program adoption? |
| Implementation | - Thank you for completing the adherence checklist - can you elaborate on the program dose - that is how much of the program was delivered as intended (where, how and why was Transform-Ed! embedded into the unit) - Frequency – how often was it delivered within the unit? - (adherence/fidelity) - Were you satisfied with delivery? - In your opinion, what are the key elements of the intervention that must be delivered to be successful? - What are the barriers and enablers to delivery? - What adaptations or modifications do you think will be necessary to help implement the initiative to fit the different settings? - What do you perceive to be the major barriers and facilitators to the scaled-up implementation of Transform-Ed! within the Bachelor of Education (primary)?   - The School of Education more broadly?   - Other universities? - What are/could be some suggested solutions to barriers? |
| Maintenance | What were the adaptions you made to the program as it moved (i) intensive and (ii) online formats?   - 1. to dose and frequency   2. delivery methods   3. key content   4. artefacts or evidence - share your thoughts of perceived effectiveness of the program across the differing versions (post-2019 trial) - Can it be sustained   - In your practice?   - in this unit?   - In other units?   - In ITE? - What is needed for this to occur? |
