# Supplementary material for: Embedding Active Pedagogies within Pre-Service Teacher Education: Implementation Considerations and Recommendations
Source: Children (Basel). 2020 Nov 2;7(11):207. doi: 10.3390/children7110207 (PMC7692750; doi:10.3390/children7110207)
Supplement: Supplementary file 1 [file children-07-00207-s001.zip › Supplementary files/Supplementary Table 5.pdf]

Supplementary Table 5: Results from linear mixed models used to assess the intervention effects on factors associated with successful implementation of active pedagogic strategies in pre-service teachers.

	Willingness					Student outcomes					Confidence in class				
Predictors	Estimate	95%CI		p-value	β	Estimate	95%CI		p-value	β	Estimate	95%CI		p-value	β
**Fixed effects**															
(intercept)	24.36	23.77	24.95	< 0.001	-0.16	23.64	22.94	24.35	< 0.001	-0.26	20.17	19.48	20.87	< 0.001	-0.16
Time (ref. Baseline)															
Follow-up	0.54	0.22	0.86	0.001	0.21	1.75	1.32	2.18	< 0.001	0.46	1.76	1.31	2.21	< 0.001	0.55
Sex (ref. Male)															
Female	0.21	-0.38	0.81	0.479	0.08	0.29	-0.40	0.99	0.411	0.08	-0.44	-1.12	0.24	0.209	-0.14
Age (ref. 17-21 years)															
22-26 years	0.78	0.15	1.42	0.016	0.30	1.48	0.74	2.23	< 0.001	0.39	0.07	-0.66	0.79	0.860	0.02
27 or older	0.21	-0.88	1.29	0.710	0.08	0.76	-0.52	2.03	0.245	0.20	-0.62	-1.87	0.62	0.328	-0.19
Year (ref. First year)															
Second year	-0.41	-1.20	0.39	0.316	-0.16	-0.12	-1.05	0.82	0.803	-0.03	-0.27	-1.19	0.64	0.558	-0.09
Third year or more	-2.92	-4.04	-1.80	< 0.001	-1.12	-8.85	-10.17	-7.54	< 0.001	-2.32	-0.88	-2.17	0.41	0.180	-0.27
Course (ref. Bachelor Ed.)															
Bachelor of Arts	0.34	-0.58	1.26	0.471	0.13	0.20	-0.88	1.28	0.720	0.05	0.42	-0.63	1.48	0.431	0.13
Other	0.81	-0.62	2.23	0.267	0.31	0.12	-1.55	1.79	0.888	0.03	1.41	-0.22	3.04	0.090	0.44
**Random effects**															
Group: Identity															
Variance (intercept)	2.64	1.94	3.58			2.88	1.92	4.32			2.35	1.44	3.83		
Variance (residual)	3.57	3.02	4.23			6.44	5.44	7.63			6.95	5.87	8.24		
ICC	0.42	0.33	0.52			0.31	0.21	0.43			0.25	0.16	0.38		

	Competence					Confidence out of class					Barriers				
Predictors	Estimate	95%CI		p-value	β	Estimate	95%CI		p-value	β	Estimate	95%CI		p-value	β
**Fixed effects**															
(intercept)	17.20	16.56	17.84	< 0.001	-0.46	17.86	17.05	18.66	< 0.001	-0.17	48.58	46.18	50.98	< 0.001	0.42
Time (ref. Baseline)															
Follow-up	2.84	2.38	3.30	< 0.001	0.85	1.72	1.15	2.29	< 0.001	0.45	-8.25	-9.73	-6.77	< 0.001	-0.73
Sex (ref. Male)															
Female	-0.19	-0.82	0.43	0.546	-0.06	-0.28	-1.06	0.50	0.479	-0.07	0.39	-1.99	2.77	0.749	0.03
Age (ref. 17-21 years)															
22-26 years	0.22	-0.45	0.89	0.523	0.07	0.13	-0.71	0.96	0.763	0.03	-0.72	-3.26	1.82	0.579	-0.06
27 or older	-1.58	-2.73	-0.44	0.007	-0.48	-1.81	-3.24	-0.38	0.013	-0.48	-1.84	-6.18	2.51	0.408	-0.16
Year (ref. First year)															
Second year	0.49	-0.34	1.33	0.248	0.15	-0.22	-1.27	0.83	0.681	-0.06	0.40	-2.79	3.59	0.805	0.04
Third year or more	0.41	-0.77	1.58	0.499	0.12	-0.39	-1.86	1.09	0.608	-0.10	-3.85	-8.34	0.64	0.093	-0.34
Course (ref. Bachelor Ed.)															
Bachelor of Arts	0.43	-0.54	1.39	0.388	0.13	0.73	-0.48	1.94	0.236	0.19	-2.89	-6.57	0.79	0.124	-0.25
Other	1.14	-0.36	2.63	0.136	0.34	1.10	-0.77	2.97	0.250	0.29	-1.78	-7.47	3.92	0.541	-0.16
**Random effects**															
Group: Identity															
Variance (intercept)	1.14	0.46	2.85			2.02	0.91	4.49			32.71	21.55	49.65		
Variance (residual)	7.50	6.34	8.89			11.28	9.52	13.36			76.52	64.60	90.63		
ICC	0.13	0.05	0.30			0.15	0.07	0.31			0.30	0.20	0.42		

Notes : N obs = 536; N groups = 268
